# Clinical Use of Intravenous Fosfomycin in Critical Care Patients in Taiwan

**DOI:** 10.3390/pathogens12060841

**Published:** 2023-06-18

**Authors:** Tzu-Ting Chen, Yun-Fu Chang, Yea-Chwen Wu

**Affiliations:** 1 Department of Pharmacy, Taipei Medical University Hospital, Taipei Medical University, Taipei 11031, Taiwan; 2 Graduate Institute of Doctor of Pharmacy, Taipei Medical University, Taipei 11031, Taiwan; b323105017@tmu.edu.tw (Y.-F.C.); chwen5678@gmail.com (Y.-C.W.)

**Keywords:** intravenous fosfomycin, urinary tract infection, efficacy, nephrotoxicity

## Abstract

This retrospective study aimed to evaluate the clinical use and side effects of fosfomycin in critically ill patients in Taiwan. Forty-two patients (mean age, 69.9 years; female, 69%) who received fosfomycin were included from a teaching hospital in Taiwan from January 2021 to December 2021. We analyzed the prescription pattern of intravenous fosfomycin and evaluated patient safety profiles, clinical successes, and microbiological cure rates. The main indication was urinary tract infections (35.6%), and the most frequently identified pathogen was *Escherichia coli* (18.2%). The overall clinical success was 83.4%, with one multidrug-resistant pathogen isolated from eight patients (19.0%). The average dose of fosfomycin given was 11.1 ± 5.2 g/day. The average duration of therapy was 8.7 ± 5.9 days, with a median duration of 8 days, where fosfomycin was mostly (83.3%) given in combination. Fosfomycin was given 12 hourly to a maximum number (47.6%) of cases. The incidence rates of adverse drug reactions (hypernatremia and hypokalemia) were 33.33% (14/42) and 28.57% (12/42), respectively. The overall survival rate was 73.8%. Intravenous fosfomycin may be an effective and safe antibiotic to use in combination with other drugs for empirical broad-spectrum or highly suspected multidrug-resistant infections in critically ill patients.

## 1. Introduction

Fosfomycin has a unique bactericidal chemical structure that inhibits cell wall synthesis in both Gram-positive cocci (GPC) and Gram-negative bacteria (GNB) [1]. It was first discovered to belong to the phosphonic acid class of drugs in the 1960s. Intravenous (IV) fosfomycin disodium has been used as a salvage therapy for multidrug-resistant (MDR) bacterial infections, either as a monotherapy or combination therapy [1]. The COVID-19 pandemic has increased the incidence of antimicrobial resistance, especially in urinary tract infections (UTIs). Furthermore, the unique structure of fosfomycin also makes it a good alternative therapy for patients with a history of antibiotic allergy [2]. There are two fosfomycin formulae, oral and IV, with good penetration properties for complicated, biofilm-related infections with pneumonia, bone infections, central nervous system (CNS) infections, complicated urinary tract infections (cUTIs) (e.g., pyelonephritis), and complicated intra-abdominal infections (cIAI) [3,4]. Oral fosfomycin has been recommended for extended-spectrum β-lactamase-producing (ESBL) *Escherichia coli* cystitis in the Infectious Diseases Society of America 2022 Guidance [5]. The use of oral fosfomycin was restricted to cystitis due to the inability to achieve effective concentrations in other infection sites, in which IV fosfomycin would provide better penetration. The IV administration of fosfomycin was widely approved by the European Medicines Agency but less so by the Food and Drug Administration (FDA). In Europe, the use of IV fosfomycin is a good candidate for a combination of community- (CAP) and hospital-acquired pneumonia (HAP)/ventilator-associated pneumonia (VAP) in critically ill patients [6]. In Taiwan, IV fosfomycin was not used as the traditional empirical therapy because of the lack of routine MIC testing. When critically ill patients have resistant risk factors (resistant pathogen colonization, recent broad-spectrum antibiotic use), chronic kidney disease (CKD), or acute kidney injury (AKI), IV fosfomycin is considered for salvage therapy in our hospital. The most common side effect of fosfomycin is hypernatremia or hypokalemia, as 1 g of fosfomycin contains 14.5 mEq of sodium [7]. We wish that we could routinely perform fosfomycin susceptibility testing. Fosfomycin susceptibility testing is currently not included in the standard pathogen test at the Clinical and Laboratory Standards Institute. Thus, we want to implement the fosfomycin medication use evaluation (MUE). However, no such studies have been conducted in the Taiwanese critical care population for severe infections. Currently, the clinical use, microbiological efficacy, and safety of fosfomycin in critically ill patients are limited. This study aims to reveal real-world data on IV fosfomycin use in critical care units in Taiwan.

## 2. Materials and Methods

### 2.1. Study Design and Population

This was a retrospective single-center study in which the analyzed data showed the medical records of 42 patients admitted to the intensive care unit of Taipei Medical University Hospital (TMUH) from 1 January to 31 December 2021. Our inclusion criteria were hospitalized adult patients (aged 20–100 years) treated with fosfomycin for various infections who received at least 48 h of fosfomycin disodium. Patients with missing data and those who died less than 24 h after the first dose were excluded. Patient medical records were obtained by reviewing microbiology reports and bacteremia databases. The reconstitution and final dilution of fosfomycin (YUNH SHIN PHARMACEUTICAL INDUSTRIAL CO., LTD, Taiwan) was within 10–40 mg/mL using 5% glucose because of the high sodium content, which may warrant caution for patients with heart failure. The dose range of fosfomycin is shown in Table 1.

### 2.2. Data Collection and Measurements

Table 2 shows the patient data, including age, ward type, definite diagnosis, weight, serum creatinine (SCr), medical history (hospitalization history, broad-spectrum antibiotic within 90 days), fosfomycin dose and duration, isolated pathogens, MDR type, laboratory parameters, serum electrolytes (sodium and potassium), liver enzymes, urinalysis, and assessment of adverse or serious adverse events before and after fosfomycin treatment. Concomitant antimicrobial agents and microbiological, culture, and susceptibility tests were also collected.

### 2.3. Definitions

**Figure 1:**
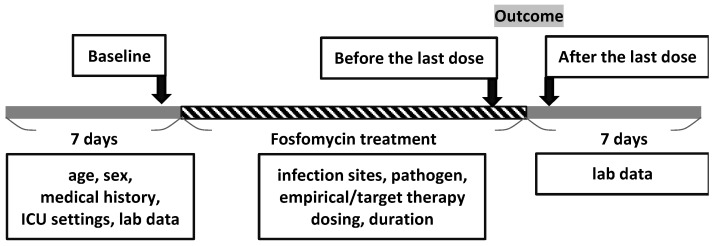
Timeline of fosfomycin treatment.

The timeline of fosfomycin treatment is shown in Figure 1. The doses of fosfomycin, number of days for which fosfomycin therapy was administered, antimicrobial pretreatment, concomitant use of other antimicrobial agents, aspartate aminotransferase (AST), alanine aminotransferase (ALT), serum sodium level (Na), serum potassium level (K), eosinophilia (EOS), urine analysis (UA), site of bacterial culture, and bacterial strains were recorded for the time period from admission (baseline), before and after the last dose within 7 days, from the patient’s medical records to capture any significant findings (Figure 1). The time and reasons for discontinuation owing to adverse events were recorded. If AST/ALT increased up to three times (>120 U/L) or the total bilirubin up to 1.2 mg/dL, it was defined as liver toxicity. Hypokalemia and hypernatremia were defined as serum levels of <3.5 mEq/L and >145 mEq/L, respectively.

Clinical cure or improvement was defined as clinical success and clinical effectiveness population. The resolution of the signs and symptoms of infection and/or no additional antibiotic therapy was defined as a clinical cure. Clinical improvement (improvement in signs and symptoms of infection) were defined as decreasing C-reactive protein (CRP) levels to half and procalcitonin levels to <0.5 after ending fosfomycin treatment. Clinical failure was defined as the administration of additional antibiotic therapy. Eradication of the underlying pathogen was defined as microbiological success and microbiological effectiveness population.

### 2.4. Outcome Analysis

We analyzed the dose and duration of fosfomycin administration as the primary outcomes. The primary efficacy endpoint was overall clinical success. The secondary efficacy endpoints included the urinary tract infection (UTI) subgroup, number of patients with a cure of infection, improvement of infection, unaltered infection, clinical treatment failure, pathogen eradication, and microbiological treatment failure. Secondary outcomes included an analysis of the risk factors related to potential adverse events in patients treated with fosfomycin.

### 2.5. Statistical Analysis

All categorical and continuous variables are expressed as proportions, means, and standard deviations (SDs). The side effects of fosfomycin, such as transaminase (AST/ALT) elevation, hypernatremia, and hypokalemia before and after IV fosfomycin, and the dose range were analyzed with two-way ANOVA and RM ANOVA using SPSS software (yongxi-stat, Taipei, Taiwan).

## 3. Results

### 3.1. Patients’ Characteristics

Between January 2021 and December 2021, 42 patients who received fosfomycin were identified. None of the patients were excluded from the study. Patient demographics and clinical characteristics are presented in Table 2. The mean patient age was 69.9 ± 15.3, with 83.3% admitted to the surgical intensive care unit and 69% female patients. Pre-exposure to broad-spectrum antibiotics and hospitalization within 3 months were 40.5% and 28.6%, respectively, whereas 64.3% (*n* = 27) of patients had a history of surgery. Most patients (83.3%) had received fosfomycin as an empirical therapy, and only 14.3% (*n* = 6) received empirical targeted therapy without repeat culture after antibiotics. The average weight-based daily dose and the daily dose of fosfomycin were 160.3 ± 76.7 mg/kg/day and 11.1 ± 5.2 g/day, respectively. The highest proportion of daily dose (42.9%) was 12–16 g/day. The treatment duration of fosfomycin therapy was 8.7 ± 5.9 days, with a median duration of 8 days. The percentage of patients in whom fosfomycin was administered as combination therapy against resistant Gram-positive and Gram-negative bacteria was 2.4% (*n* = 1) and 16.67% (*n* = 7), respectively (Table 2).

### 3.2. Description of the Infection and Microbiological Data

The top three indications for fosfomycin were cUTI (*n* = 16, 35.6%), bacteremia (*n* = 7, 15.6%), and intraabdominal infection (*n* = 7, 15.6%) (Table 3). The pathogens found in the culture were Gram-negative (22 isolates, 50.0%) and Gram-positive (11 isolates, 25.0%), as shown in Table 4. No bacteria or other definite cultures could be identified in 13 (29.5%) patients. The full microbiological spectrum of the 33 isolates is shown in Table 5, with the most frequently identified pathogens being *E. coli* (18.2%), *Pseudomonas aeruginosa* (12.1%), *Staphylococcus aureus* (12.1%), *Enterococcus faecalis* (12.1%), and *Klebsiella pneumoniae*. (9.1%); 9/33 (27.3%) MDR pathogens were isolated, and 8/9 (88.9%) MDR pathogens were treated by combination therapy. Fosfomycin was mostly used in combination therapy (*n* = 35, 83.3%) and predominantly combined with a β-lactam + β-lactamase inhibitor (BL + BLI), carbapenem, or a 3rd- or 4th-generation cephalosporin (Table 6). The average clinical success rate of the fosfomycin combination regimen in subgroups of patients with bacterial pneumonia, abdominal infection, and CNS infection (all without sepsis or bacteremia) was 87.5% (Table 7). One of three patients with bacterial pneumonia (CAP, HAP, or VAP) was prescribed empirical meropenem combined with IV fosfomycin, followed by de-escalation to cefepime for target therapy with IV fosfomycin. In the abdominal infection group, one of seven patients received empirical IV fosfomycin monotherapy and had been defined as a clinical failure. In the UTI infection group, one of sixteen patients received carbapenem, a 3rd- or 4th-generation cephalosporin, and BL + BLI with IV fosfomycin combination therapy, while four of sixteen patients received IV fosfomycin monotherapy; the clinical success rate was 75%. In the SSTI infection group, two of five patients received empirical IV fosfomycin monotherapy, and the clinical success rate was 100% (Table 7).

### 3.3. Secondary Outcome

Overall survival was observed in 73.8% of patients (31/42), and the clinical success rate was favorable in 83.4% (35/42) of cases. The microbiological successful eradication rate was 21.4%, whereas the failure rate was 2.4% (Table 8). Moreover, the major side effects of fosfomycin were hypernatremia (14/42, 33.33%) and hypokalemia (12/42, 28.57%). However, the incidences of hypernatremia and hypokalemia were not significantly affected by post-exposure time (*p* > 0.05 and >0.05, respectively) or dose dependence (*p* > 0.05 and >0.05, respectively) in the two-way ANOVA. In contrast, hypokalemia incidence was significantly affected between the time of IV fosfomycin treatment initiation and the time of its discontinuation, according to RM ANOVA (*p* < 0.001). The increase in transaminases (AST/ALT) was minor, around 3%, without significant dose-dependent or related renal function (*p* > 0.05 and >0.05, respectively, on the two-way ANOVA) (Table 9).

### 3.4. Secondary Analysis Based on Subgroups

Of the 42 patients, 16 received fosfomycin treatment for UTI. The demographic data, fosfomycin dosage, UTI pathogens, and characteristics of these patients, such as the dosage, clinical, and microbiological efficacy endpoints, were analyzed and are presented in Table 10 and Table 11. Only one of the three MDR UTI regimens was IV fosfomycin monotherapy. Of the sixteen patients (38%), nine had received moderate to high doses of fosfomycin (daily dose 8–12 g/day), three had MDR pathogens, and the clinical efficacy endpoint was up to 80–100%. Overall, the cure rate of fosfomycin in cUTI treatment was 68.8% (11/16) of cases, and the clinical failure rate was 12.5% (a carbapenem-resistant *K. pneumoniae* (CRKP) cUTI and an *E. faecalis* catheter-related cUTI). The microbiological successful eradication rate was 12.5%, whereas the failure rate was 6.3% (Table 12). Fosfomycin for cUTI was nearly always used in combination therapy (14 patients, 87.5%) and predominantly combined with a BL + BLI (50%). The clinical efficacy endpoint for BL + BLI combination therapy was up to 87.5%, as only one patient (12.5%) with CRKP had failed treatment (Table 13).

## 4. Discussion

### 4.1. Patient Characteristics

MDR infections are a huge challenge for clinically available antimicrobial agents and the economic outcomes of patients. Combination therapy can provide broad empirical coverage using agents with different mechanisms of action against MDR pathogens [8,9]. In this retrospective observational study, we evaluated the prescription pattern and dosage of IV fosfomycin and the correlation between dose-dependent side effects, clinical outcomes, and microbiological efficacy.

The prescription pattern in our study was consistent with that in other studies; however, *E. coli* was the most common pathogen that fosfomycin was used against in our study, while fosfomycin was prescribed more frequently for *K. pneumoniae* infections in another study [10]. The properties of fosfomycin that contribute to its efficacy include a high plasma concentration, tissue penetration, low cross-resistance, and low renal toxicity [1].

### 4.2. Deep GPC Infection

Despite being unable to evaluate IV fosfomycin dosing regimens in critically ill patients with carbapenem-resistant *Enterobacterales* (CRE) infections, fosfomycin may be beneficial for the treatment of difficult-to-treat deep GPC infections, such as *S. aureus* and *Staphylococcus epidermidis* infections [4,7]. In vancomycin-resistant *Enterococcus faecium* bacteremia treatment, fosfomycin in combination with high-dose daptomycin showed low mortality. In a 2022 prospective observational multicenter study by Shan-Chwen et al., the fosfomycin minimum inhibitory concentration (MIC) was 64 mg/L in 70.8% of the isolates [11]. We had three GPC osteomyelitis cases with 100% clinical improvement and 66.7% microbiological eradication when fosfomycin was combined with penicillin.

### 4.3. Difficult-to-Treat Resistance GNB Infection

Regarding Gram-negative pathogens, including MDR bacteria, such as extended-spectrum beta-lactamase (ESBL)-producing and/or carbapenemase-producing enterobacteria, fosfomycin is active; it has less nephrotoxicity than colistin when used in combination with other drugs [12]. Litty et al. reported that the rate of fosfomycin for MDR pathogens in intensive care unit (ICU) patients was 24% [10], similar to our study’s 27.3%. The benefits of using fosfomycin in combination regimens stem from its synergistic and additive effects via different mechanisms [13]. We found the most common fosfomycin combination partners were penicillins + beta-lactamase inhibitor (38.1%) (Table 6), whereas the most common fosfomycin combination partners were carbapenem (48.8%) in the Litty et al. study [7]. In hospital-acquired infections (bacterial pneumonia (CAP, HAP, or VAP) or bloodstream infection (BSI)) (Table 7), fosfomycin was the most common combination drug used with carbapenem in our study. We encountered 14 isolate of CRE pathogens. Of these, failure was reported in three cases, one of which was due to the use of a low dose (6 g/day) of fosfomycin as monotherapy. The other cases (13/14) received over 8 g/day of fosfomycin. Leelawattanachai et al. found that a total daily dose of more than 8 g/day would be effective [14], which is consistent with the 78.6% (11/14) clinical efficacy of fosfomycin against CRE infections in our study. However, we found only one 4 g/day fosfomycin in combination with meropenem for carbapenem-resistant *P. aeruginosa* that was effective.

During the COVID-19 pandemic, the increasing consumption of antibiotics accelerated the development of drug-resistant microorganisms, especially those causing UTIs. In Romanian patients, the most common uropathogen was *E. coli*, which exhibited up to 72.08% and 66.78% resistance to quinolones and penicillin, respectively [15,16,17]. Dimopoulos et al. (2019) reported an IV fosfomycin dose range of 12–24 g/day, which showed maximal efficacy with minimal toxicity in observational non-comparative trials [18]. In a real-world prescription pattern critical care patient study, a daily dose of more than 12 g of fosfomycin could achieve better clinical outcomes for *E. coli* UTI [9]. In our study, 43.8% of patients (*n* = 7) with GNB UTIs were treated with fosfomycin, and 42.8% (*n* = 3) had MDR infections. In our study, a daily dose of more than 8 g of fosfomycin could attain 80–100% clinical efficacy, although we did not use the fosfomycin MIC test. In the Jesús Rodríguez Randomized Clinical Trial, fosfomycin did not demonstrate non-inferiority to comparators as a targeted treatment of UTI with MDR *E. coli* [19]. Our study found that if MDR *P. aeruginosa, K. pneumoniae,* and pan-resistant *Chryseobacterium indologenes* cUTIs were treated with a combination of fosfomycin and BL + BLI or carbapenem, the clinical efficacy success rate was up to 87.5%. Only one patient (33%) with a CR-*K. pneumoniae* UTI under fosfomycin monotherapy experienced microbiological and clinical failure.

### 4.4. Fosfomycin Safety

Critically ill patients with acute renal failure and complicated underlying diseases face a huge clinical challenge for the optimization of effective and safe antibiotic therapy. Nephrotoxic medications (e.g., aminoglycoside, vancomycin, or colistin) are avoided as combination therapy during the acute renal failure phase to protect patients’ renal function. However, IV fosfomycin has good penetration, a wide therapeutic range, and less severe side effects (hypernatremia and hypokalemia without renal toxicity) [20], making it a good candidate for severe infection control. In our study, the incidence of fosfomycin as an alternative renal protection therapy was approximately 43%.

The European Medicines Agency had announced to clinicians that IV fosfomycin should be used with caution in patients with heart failure. During IV fosfomycin therapy, the serum sodium and potassium levels should be monitored to prevent complications [21].

This study had several limitations. For example, the sample size obtained during one year was small, and the interval between blood collections for serum electrolyte level measurement and the interval between specimen collections was inconsistent. The Infectious Diseases Society of America does not recommend repeat cultures within 5 days of a positive urine culture. Given the lack of sensitivity of fosfomycin, we cannot consider it the only active drug or the effective combination, which was contributed by fosfomycin exactly. Furthermore, the clinicians did not recheck the sputum culture if there were no signs of pneumonia. Therefore, the microbiological efficacy could not be determined. Other causes of electrolyte imbalance may have been underestimated, and the concomitant use of medications for other causes of liver injury was not discussed in the analysis of liver toxicity. We hope that future studies can overcome these limitations and offer optimized analyses.

## 5. Conclusions

We found that IV fosfomycin was an effective and safe candidate in critically ill patients infected with GPC and GNB strains treated with combination therapy, with a high clinical success rate of 83.4%. Hence, these findings support the use of moderate-to-high doses of fosfomycin in Taiwan, even without MIC data.

## Figures and Tables

**Table 1:** The fosfomycin dosing guide.

Infection Status Renal Clearance (mL/min)	Mild	Moderate	Severe
>50	2 g q8 h	4 g q6 h	6–8 g q8 h
10–50	2 g q12 h	8 g/day divided q6–12 h	12–16 g/day divided q6–8–12 h
<10	2 g q24 h	4 g/day divided q12–24 h	6–8 g/day divided q6–8–24 h
Hemodialysis (HD)	2 g q8 h/8 g q24 h (after HD)		
Continuous renal replacement therapy (CRRT)	8 g q12 h		

**Table 2:** Patients’ demographic and clinical characteristics.

Characteristic		Baseline (*n* = 42)
Age, years (mean, SD)		69.9 ± 15.3
Gender, female (n, %)		29 (69%)
Weight, kg (mean, SD)		72.4 ± 15.4
Scr, mg/dL (mean, SD)		2.3 ± 2.0
ClCr, mL/min (mean, SD)		52.9 ± 44.8
Hospitalization within 3 months (n, %)		12 (28.6%)
Broad-spectrum antibiotics within 3 months (n, %)		17 (40.5%)
Surgery (n, %)		27 (64.3%)
ICU setting (n, %)		
- EICU		2 (4.8%)
- SICU		35 (83.3%)
- MICU		5 (11.9%)
Empirical therapy with fosfomycin (N, %)		35 (83.3%)
Target therapy with fosfomycin (N, %)	Pathogen (positive)	16 (38.1%)
	Pathogen (negative)	6 (14.3%)
Daily dose of fosfomycin, g/day (mean, SD)		11.1 ± 5.2
Weight-based daily dose, mg/kg/day (mean, SD)		160.3 ± 76.7
Fosfomycin dosing (gm/day)		
4		4 (9.5%)
6		4 (9.5%)
8		12 (28.6%)
12–16		18 (42.9%)
>16–24		4 (9.5%)
Treatment duration of fosfomycin, day (mean, SD)		8.7 ± 5.9
Combination for MDR GNB		7 (16.67%)
Combination for MDR GPC		1 (2.4%)

**Table 3:** Source of infection.

Infection Site (*n* = 42)	Number *n* = 45 (%)
UTI	16 (35.6)
Bacteremia	7 (15.6)
IAI	7 (15.6)
SSTI	5 (11.1)
Pneumonia	4 (8.9)
Osteomyelitis	3 (6.7)
Endocarditis	1 (2.2)
Sepsis unknown infection	2 (4.4)

UTI: urinary tract infection; IAI: intra-abdominal infection; SSTI: skin-soft tissue infection.

**Table 4:** Culture results.

Culture Results (*n* = 46) **	Number (*n* = 46) (%)
GPC	11 (25.0)
GNB	22 (50.0)
No Pathogen	13 (29.5)

GPC: Gram-positive cocci; GNB: Gram-negative bacteria; ** intra-abdominal infection culture results were multiple pathogens. *n* > 42.

**Table 5:** Most frequently identified pathogens.

Pathogens	
GNB	*n* = 22 (%)
*Escherichia coli*	6 (18.2)
*Pseudomonas aeruginosa*	4 (12.1)
*Klebsiella pneumoniae*	3 (9.1)
*Enterobacter cloacae complex*	2 (6.1)
*Acinetobacter baumannii*	1 (3.0)
*Chryseobacterium indologenes*	1 (3.0)
*Citrobacter freundii*	1 (3.0)
*Enterobacter aerogenes*	1 (3.0)
*Klebsiella oxytoca*	1 (3.0)
*Serratia marcescens*	1 (3.0)
*Proteus mirabilis*	1 (3.0)
GPC	11
*Staphylococcus aureus*	4 (12.1)
*Enterococcus faecalis*	4 (12.1)
*Enterococcus faecium*	1 (3.0)
*Streptococcus agalactiae (Strep. group B)*	1 (3.0)
*Streptococcus anginosus*	1 (3.0)
No pathogen	13

**Table 6:** Fosfomycin combination partners with number of patients per class (*n* = 35).

Combination Partners	Number of Patients (n, %)
Penicillins + beta-lactamase inhibitor	16 (38.1)
Carbapenem	10 (23.81)
3rd- or 4th-generation cephalosporin	7 (16.67)
Penicillin	5 (11.90)
Glycopeptide	2 (4.76)
Tetracyclines	2 (4.76)
1st- or 2nd-generation cephalosporin	1 (2.38)
Lincomycins	1 (2.38)
Anti-fungal	3 (7.14)
None	2 (4.76)

**Table 7:** Fosfomycin combination partners in subgroups of patients with bacterial pneumonia, abdominal infection, and CNS infection (all without sepsis or bacteremia).

Combination Partners	Number of Patients (%) (Total Combination Partners, *n* = 35)	Clinical Effectiveness Population	Microbiological Effectiveness Population
Patients with bacterial pneumonia (CAP, HAP, or VAP)	3 (100)		
Carbapenem	2 (66.7)	2 (100%)	
3rd- or 4th-generation cephalosporin	1 (33.3)	1 (100%)	
β-lactam + β-lactamase inhibitor (BL + BLI)	1 (33.3)	0 (0%)	
Patients with abdominal infection	7 (100)		
β-lactam + β-lactamase inhibitor (BL + BLI)	3 (42.9)	3 (100%)	1 (33.33%)
3rd- or 4th-generation cephalosporin	2 (28.6)	2 (100%)	
Carbapenem	1 (14.3)	1 (100%)	
Fosfomycin monotherapy	1 (14.3)	0 (0%)	
Patients with UTI infection	16 (100)		
β-lactam + β-lactamase inhibitor (BL + BLI)	8 (50.0)	1 (87.5%)	1 (12.50%)
3rd- or 4th-generation cephalosporin	3 (18.8)	3 (100%)	2 (66.67%)
Carbapenem	2 (12.5)	2 (100%)	1 (50.00%)
2nd-generation cephalosporin	1 (6.3)	1 (100%)	
Fosfomycin monotherapy	4 (25)	3 (75%)	
Patients with BSI infection	6 (100)		
Carbapenem	4 (71.4)	3 (75%)	3 (75.00%)
Penicillin	2 (28.6)	1 (50%)	1 (50.00%)
Patients with SSTI infection	5 (100)		
β-lactam + β-lactamase inhibitor (BL + BLI)	2 (40.0)	2 (100%)	
Glycopeptide	1 (20.0)	1 (100%)	
Fosfomycin monotherapy	2 (40)	2 (100%)	
Patients with osteomyelitis infection	3 (100)		
Penicillin	3 (100)	3 (100%)	2 (66.67%)

**Table 8:** Efficacy endpoints.

Clinical Efficacy Endpoints	*n* = 42 (%)
Cure	17 (40.5)
Improvement	18 (42.9)
Failure	3 (7.1)
Unchanged	3 (7.1)
Not assessable *	1 (2.4)
Microbiological efficacy endpoint	*N* = 42 (%)
Eradication	9 (21.4)
Microbiological treatment failure	1 (2.4)
Not reported	17 (40.5)
Not determined	11 (26.2)
Not assessable **	4 (9.5)

* The patient was died on lung edema when initiating IV fosfomycin. ** The Infectious Diseases Society of America does not recommend repeat cultures within 5 days of a positive urine culture and recheck the sputum culture without signs of pneumonia.

**Table 9:** The frequency of fosfomycin side effects.

Adverse Effects	Before the Last Dose		After the Last Dose	
	Total N	Frequency	Total n	Frequency
Aspartate aminotransferase (AST) elevation (>3 × ULN)	36	1 (2.8%)	32	2 (6.3%)
Alanine aminotransferase (ALT) elevation (>3 × ULN)	31	0 (0%)	28	2 (7.1%)
Total bilirubin elevation (>1.2 mg/dL)	28	3 (10.7%)	24	2 (8.3%)
Hypernatremia (>145 mEq/L)	42	14 (33.33%)	41	12 (29.27%)
Hypokalemia (<3.5 mEq/L)	42	12 (28.57%)	41	10 (24.39%)
Eosinophilia (>500/mcL)	39	3 (7.69%)	37	1 (2.7%)

**Table 10:** The demographic and fosfomycin dosage and clinical efficacy endpoint.

Fosfomycin Dosing (gm/Day)	Number of Patients (*n* = 16, %)	Efficacy Endpoint (n, %)	MDR Pathogen (n, %)	MDR Pathogen Efficacy Endpoint (n, %)
High dose (12–18 g/day)	4 (25)	Cure (4, 100%)	1 (25)	1 (100)
Moderate dose (8 g/day)	5 (31.2)	Cure (3, 60%) Improvement (1, 20%) Failure (1, 20%)	1 (20)	1 (100)
Low dose (4–6 g/day)	4 (25)	Cure (2, 50%) Improvement (2, 50%)		
HD	2 (12.5)	Cure (1, 50%) Failure (1, 50%)	1 (50)	0 (0)
CVVH	1 (6.3)	Cure (1, 100%)		

**Table 11:** UTI most frequently identified pathogens.

Pathogens	*n* = 16	Multidrug-Resistant (MDR) Pathogen
GNB	7 (43.75)	
*Escherichia coli*	2 (12.5)	
*Pseudomonas aeruginosa*	2 (12.5)	1
*Klebsiella pneumoniae*	2 (12.5)	1
*Chryseobacterium indologenes*	1 (6.3)	1
GPC	1 (6.3)	
*Enterococcus faecalis*	1 (6.3)	
No pathogen	8 (50)	

**Table 12:** UTI microbiological efficacy endpoints (*n* = 16).

Clinical Efficacy Endpoint	*n* = 16
Cure	11 (68.8)
Improvement	3 (18.8)
Failure	2 (12.5)
Microbiological efficacy endpoint	N = 16
Eradication	2 (12.5)
Microbiological treatment failure	1 (6.3)
Not reported	10 (62.5)
Not determined	3 (18.8)

**Table 13:** Fosfomycin combination UTI partners with number of patients per class (*n* = 16).

Patients with UTI Infection	16 (100)	Clinical Efficacy Endpoint
β-lactam + β-lactamase inhibitor (BL + BLI)	8 (50.0)	Cure: 6 (75%) Clinical improvement:1 (12.5%) Failure: 1 (12.5%)
3rd- or 4th-generation cephalosporin	3 (18.8)	Cure: 3 (100%)
Carbapenem	2 (12.5)	Clinical improvement: 2 (100%)
2nd-generation cephalosporin	1 (6.3)	Cure: 1 (100%)

## Data Availability

Due to privacy restrictions, the data presented in this study are available on request from the corresponding author.
